# An Unusual Method for Controlling Bile Leak from an Immature T-tube Fistula

**DOI:** 10.7759/cureus.2560

**Published:** 2018-05-01

**Authors:** Murat F Ferhatoglu, Sadik A Uyanik, Ali I Filiz

**Affiliations:** 1 General Surgery, Okan University Medical Faculty, Istanbul, TUR; 2 Radiology, Okan University Medical Faculty, Istanbul, TUR

**Keywords:** bile leak, coil embolization, fistula, t-tube

## Abstract

T-tubes have been used by surgeons for the decompression of the choledocus for over a hundred years. Errors in the operative technique and patient-related risk factors, such as immune comprehensive therapy, diabetes mellitus, steroid drug usage, T-tube morphology, T-tube material, and the T-tube extraction technique, are some of the factors that affect the risk of bile leak. In most cases, a surgical approach is required. Herein, we present a case of bile leak after T-tube removal and an unusual solution to this difficult complication.

## Introduction

T-tube usage for the drainage of the choledocus is the most frequently performed surgical procedure in the exploration of the choledocus for choledocholithiasis or for the repair of iatrogenic bile duct injuries. T-tubes passively decompress the pressure over the biliary tree to create a fibrous fistula resulting in fibrotic adhesions that cover the defect in the choledocus over time [1]. This procedure decreases the risk of bile leak. Routinely, a small amount of bile leak from the dermal ostium of the T-tube after the tube removal, but the leakage usually stops in 24 hours [2]. In rare cases, T-tubes cause a bile leak at the time of removal, which may force the surgeon to perform an urgent surgical intervention. In the current paper, we presented an unusual solution for a bile leak after a T-tube removal and aimed to identify the risk factors of choleperitonitis after T-tube removal. Written consent from the patient was obtained before preparation of this presentation.

## Case presentation

A 52-year-old man was admitted to the surgery clinic with a two-day history of mild abdominal pain radiating to the back, yellowing sclera, and dark-coloured urine. He had undergone a Billroth II procedure over 20 years ago for a distal gastric ulcer. Physical examination revealed a midline abdominal incision scar and mild tenderness in the right upper quadrant. Blood test results showed total bilirubin of 5 mg/dl and an unconjugated bilirubin of 3 mg/dl. The serum aspartate aminotransferase (AST) and alanine aminotransferase (ALT) levels were two times higher than normal. Ultrasonography revealed multiple subcentimetric gallstones confined to the thin-walled gallbladder with dilated intrahepatic ducts, 12-mm calibrated choledocus, and other organs were normal. Magnetic resonance cholangiopancreatography revealed a semi-obstructing gallstone 13-mm in diameter in the choledocus. Owing to his history of a Billroth II procedure, performing an endoscopic retrograde cholangiopancreatography (ERCP) for gallstone in the choledocus was not possible. Because of this reason, a surgical decision was taken. After a right subcostal incision, the gallbladder and the fatty tissues surrounding the choledocus were found to be oedematous. However, a cholecystectomy was performed without any complication, followed by choledocus exploration and stone extraction with a T-tube insertion. A latex, 12-foot T-tube was used in the operation. The horizontal branch of the T-tube was shortened, and the T-tube was incised to form a gutter, with a V-notch added at the junction of the two arms. The patient made an uneventful postoperative recovery and was discharged on postoperative day 5 with the T-tube spigotted and left *in situ*.

The patient's bilirubin levels done twice in six weeks were normal, and he had a problem-free recovery. T-tube cholangiogram performed after six weeks did not reveal bile duct stricture or leak. The T-tube was removed without any difficulty. The patient was discharged after six hours of uneventful observation. However, a day after the T-tube removal, the patient presented to the emergency clinic with complaints of fever, vomiting, and severe pain in the right upper quadrant. He was hypotensive (80/60 mmHg), had tachypnea (18 counts/min), and tachycardia (112 bpm). He had mild abdominal tenderness. An increase in the C-reactive protein (CRP) level and white blood cell count were observed in the blood test results. An abdominal ultrasonography showed the presence of perihepatic fluid. Fine needle aspiration revealed that the perihepatic fluid was bilious. An 8F drainage catheter was introduced into the fluid, and a 14F drainage catheter was inserted into the choledocus. Cholangiography detected a bile leak from the choledocus. The tract of the leak was embolized with eight coils during angiography (Video 1). After filling the tract with multiple coils and percutaneous drainage of the perihepatic fluid, the patient’s symptoms subsided, and his test results were found to be normal. The T-tube was removed on the fifth day of coiling and the percutaneous catheter was removed after a control ultrasonography on the sixth day of coiling. No perihepatic or abdominal liquid was detected on control ultrasonography on day 14 after removal of the percutaneous catheter. The patient showed no further complications at a three-month follow-up.

**Video 1:** Coil embolisation of a T-tube tract causing a bile leak

## Discussion

The first T-tube usage for bile duct decompression was described by a German surgeon, Hans Kehr, in 1897 [1]. He placed a rubber T-shaped drain into the choledocus and exteriorised the ascending limb from the abdominal wall. Next, he located one side of the descending T-tube section passing through the duodenal papilla into the descending part of the duodenum. Because the transpapillary part of the tube causes pancreatitis and forms an external fistula between the duodenum and the skin, this technique was abandoned over time [3]. Two major techniques are performed for patients with common bile duct gallstones: cholecystectomy with choledocus exploration and a combination of cholecystectomy with endoscopic clearing of the choledocus with or without sphincterotomy. Despite the developments in radiodiagnostic and endoscopic techniques, we are still unable to diagnose some of the patients who have common bile duct gallstones before surgery [4].

After the choledocus exploration, surgeons can close the defect in the choledocus in the following five ways:

     i.         Primary closure without drainage

     ii.         Suturation with internal drainage via an endoprothesis

    iii.         Suturation with transcystic ductal drainage

    iv.         Suturation with an external drainage via a T-tube

     v.         Biliary-enteric anastomosis

Routine use of T-tubes for bile drainage is associated with prolonged hospitalisation and increased care costs. Gurusamy et al. suggested that if the choledocus is not significantly dilated, choledochoraphy is as safe as T-tube drainage [5]. We preferred T-tubes instead of primary closure because suturing inflammatory and oedematous tissue may result in suture dissociation and bile leak.

In the past two decades, owing to technological advancements in radiodiagnostic-interventional radiology, new treatment modalities have gained attention for vascular lesions, such as coil or glue embolization or stent grafting. The main concept of these methods is blocking the leak with foreign materials. The use of foreign materials for treating bile leak may be a new and promising treatment modality for this challenging and expensive complication.

## Conclusions

New developments in tools of radiodiagnostic-interventional radiology have ushered in a new era in the treatment of many complications. Coil embolization may be a promising new method for treating a bile leak that is associated with prolonged hospitalisation and increased care costs. For a better evaluation and validation of this treatment approach, further research with a large case series is warranted.
